# Effects of canagliflozin on kidney resistive index and oxygenation in patients with type 2 diabetes: findings obtained with ultrasonography and blood oxygenation level-dependent MRI

**DOI:** 10.3389/fcdhc.2026.1733806

**Published:** 2026-04-29

**Authors:** Hideki Uedono, Katsuhito Mori, Tsutomu Inoue, Yuri Machiba, Fumiyuki Morioka, Shinya Nakatani, Akihiro Tsuda, Hirokazu Okada, Masanori Emoto

**Affiliations:** 1 Department of Metabolism, Endocrinology and Molecular Medicine, Osaka Metropolitan University Graduate School of Medicine, Osaka, Japan; 2 Department of Endocrinology, Metabolism and Diabetes, Kindai University Nara Hospital, Nara, Japan; 3 Department of Nephrology, Faculty of Medicine, Saitama Medical University, Saitama, Japan; 4 Department of Nephrology, Osaka Metropolitan University Graduate School of Medicine, Osaka, Japan

**Keywords:** BOLD MRI, canagliflozin, kidney oxygenation, resistive index, SGLT2 inhibitor

## Abstract

**Background:**

Details regarding kidney-protective mechanisms of sodium-glucose cotransporter 2 (SGLT2) inhibitors remain unclear. High kidney resistive index (RI), evaluated by Doppler ultrasonography, and low T_2_* value, evaluated by blood oxygenation level-dependent (BOLD) magnetic resonance imaging (MRI) are risk factors for chronic kidney disease progression. In our previous study, canagliflozin (Cana) improved kidney oxygenation. In this *post-hoc* analysis, the effects of Cana on RI, as well as the correlation between changes in RI and T_2_* values, were evaluated.

**Methods:**

Thirteen patients with type 2 diabetes (T2D) were analyzed. RI was determined at screening and on Day 5 (D5) following administration of Cana, while BOLD MRI was performed on D0, D1 (administration day), and D5. Cortical T_2_* values were evaluated using twelve-layer concentric object (TLCO) and region of interest (ROI) methods. Changes in RI and T_2_* values from D0 to D1 or D5 are expressed as ΔRI, ΔT_2_* (D1–D0), and Δ T_2_* (D5–D0), respectively.

**Results:**

Cana significantly decreased RI, while ROI findings showed increased T_2_* (D0: 54.5 [50.8–55.5] vs D1: 56.2 [52.7–56.4], *p* = 0.003, *r* = 0.77; D0 vs D5: 54.5 [53.0–57.2], *p* = 0.080, *r* = 0.49). A significant negative correlation between ΔRI and ΔT_2_* (D1–D0) was shown by both TLCO (*ρ* = -0.621, *p* = 0.024) and ROI (*ρ* = -0.557, *p* = 0.048).

**Conclusion:**

Short-term Cana treatment improved RI and kidney oxygenation in patients with T2D. The inverse correlation found between changes in RI and T_2_* values suggest a mechanistic link between hemodynamic changes and improved oxygenation by SGLT2 inhibitors.

## 1 Introduction

Recent large-scale clinical trials have demonstrated beneficial effects of sodium-glucose cotransporter 2 (SGLT2) inhibitors on kidney outcomes (1–4). Proposed mechanisms include improvements in glomerular hyperfiltration and altered energy metabolism, notably through increased ketone body utilization (5), though several underlying factors remain unclear.

Kidney resistive index (RI), determined by pulsed-wave Doppler ultrasonography of renal interlobar arteries, reflects reduction in end-diastolic blood flow velocity relative to peak systolic velocity. While considered to be an indicator of peripheral vascular resistance, recent studies have also suggested that RI may reflect systemic vascular impedance and reversible renal hemodynamic changes (6). Studies have shown elevated RI in patients with diabetic kidney disease (DKD) (7), chronic kidney disease (CKD), and renal artery stenosis (8). RI has also been identified as a predictor of all-cause mortality in heart failure (9) and adverse kidney outcomes in DKD (10).

Blood oxygen level-dependent magnetic resonance imaging (BOLD MRI) has become recognized as a noninvasive method for evaluating renal tissue oxygenation. Differences in magnetic susceptibility between oxygenated and deoxygenated hemoglobin lead to T2 signal changes (BOLD effect). Studies using BOLD MRI have shown that higher R2* (11) and lower T2* (12) (indicative of poor oxygenation) values are associated with increased risk of CKD progression.

Doppler ultrasonography findings have shown that SGLT2 inhibitors improve RI in patients with type 2 diabetes (T2D) (13). Additionally, a single dose of dapagliflozin was reported to significantly reduce R2* in patients with type 1 diabetes (14), while 24 weeks of canagliflozin (Cana) treatment significantly increased T_2_* in those newly diagnosed with T2D (15). In contrast, it has also been reported that 32 weeks of empagliflozin alone, as well as empagliflozin in combination with semaglutide, may even reduce kidney oxygenation (16). Although these studies included patients with preserved kidney function, we previously reported, using BOLD MRI, that Cana improved kidney oxygenation in patients with T2D, including those with kidney impairment (17). Nevertheless, the relationship between Cana-induced changes in RI and T_2_* values remains unclear. Therefore, the present study was conducted to investigate the effects of Cana on RI and the correlation between Cana-induced changes in RI and T_2_* values in relation to hemodynamics and oxygenation.

## 2 Research design and methods

### 2.1 Study design

This study was conducted as a *post-hoc* analysis of findings obtained in a single-arm interventional study to evaluate the effects of Cana on kidney oxygenation shown by BOLD MRI findings in patients with T2D (17). Changes in RI were determined using ultrasonography findings and analyzed. The protocol for this original study was reviewed and approved by the Certified Review Board of Osaka Metropolitan University Hospital (no. CRB52000004) (Osaka Diabetes Mellitus and Kidney Diseases study-8: Diamond study-8). This trial was registered in the Japan Registry of Clinical Trials (jRCTs051200047), and conducted in accordance with the principles of the Declaration of Helsinki and Ethical Guidelines for Medical and Biological Research Involving Human Subjects issued by the Ministry of Health, Labor and Welfare, Japan (March 2021). The first patient was enrolled on September 7, 2020 and the final patient was discharged on March 2, 2022.

### 2.2 Subjects

The subjects were recruited from outpatients undergoing treatments at Osaka Metropolitan University Hospital. Inclusion criteria included T2D diagnosis, age 20–80 years, HbA1c level ≥6.5% and <10%, and no use of an SGLT2 inhibitor for at least two weeks. Exclusion criteria were as follows: 1) pregnant or currently breastfeeding, 2) known allergy to SGLT2 inhibitor, 3) contraindications for MRI including presence of pacemaker or claustrophobia, 4) history of recurrent urinary and/or genital infections, 5) nephrotic syndrome, 6) use of diuretics and/or non-steroidal anti-inflammatory medications, 7) severe kidney impairment (eGFR<30 mL/min/1.73 m^2^), dialysis dependence, or history of kidney transplantation, 8) abnormal renal morphology, such as multiple renal cysts, hydronephrosis, or severe calcification, 9) severe liver dysfunction, defined as aspartate aminotransferase (AST) or alanine aminotransferase (ALT) level three times above the upper limit of normal according to institutional standards, 10) malignant tumor history, 11) severe heart failure, 12) participation in another clinical study, and 13) considered inappropriate for study participation by primary physician. All subjects received a detailed explanation of the study objectives and provided informed written consent. Following consent, a screening visit was conducted, which included a medical history review, physical examination, blood and urine testing, and kidney ultrasonography to confirm eligibility based on the inclusion and exclusion criteria.

### 2.3 Procedures

The study schedule is summarized in **Supplementary Figure S1**. After obtaining informed consent, kidney ultrasonography was performed during the screening visit prior to hospitalization to assess baseline RI. All enrolled subjects were subsequently hospitalized to minimize potential confounding effects of sodium intake, calorie intake, and body fluid volume on kidney oxygenation, as described in our original study (17). Dietary intake was standardized to approximately 25–30 kcal/kg of ideal body weight, with 60% of the calories derived from carbohydrates, and daily salt intake of 6–7 g. To maintain hydration, subjects consumed 350 mL of water within approximately 1 hour prior to each BOLD MRI examination. The imaging protocol followed that of the original study. BOLD MRI was initially performed on the evening of the admission day (Day -2) to obtain baseline findings. Additional baseline scans, including T2-weighted imaging (T2WI) and diffusion-weighted imaging (DWI), were conducted 2 days following admission (Day 0). On Day 1, each subjects received a single oral dose of Cana (100 mg) at 2 ± 1 hours before the BOLD MRI examination, based on pharmacokinetic data indicating a peak plasma concentration approximately 1 hour after administration. Following Day 1, the subjects received Cana each morning. The fourth and final BOLD MRI examination was performed on Day 5, after five consecutive days of treatment, as steady-state plasma concentrations of Cana have been reported to be achieved within 4 days after administration (18). On Day 5, to evaluate correlations between changes in RI and kidney oxygenation, a second RI measurement was performed concurrently with BOLD MRI.

### 2.4 Doppler ultrasound

The characteristic Doppler waveform morphology for intrarenal arteries shows a steep systolic upstroke followed by a descending wave representing the diastolic component (6). RI was calculated using the following formula:


RI= (peak systolic velocity – end diastolic velocity)/peak systolic velocity

Doppler measurements were obtained using an ultrasound diagnostic system (APLIO 500, Toshiba Medical Systems, Tokyo, Japan). To ensure accuracy and reproducibility, those were obtained from multiple regions of both kidneys, with the final RI determined as the average of these measurements. Additionally, to reduce intra-observer variability, each measurement was performed at least three times. Excessive pressure with the ultrasound probe was avoided, as that can alter the Doppler signal, resulting in inaccurate RI. Measurements were performed by two independent operators, and the final RI value was defined as the average of the values obtained by the two operators. All imaging analyses were performed blinded to clinical data.

### 2.5 MRI examinations

BOLD MRI was performed using an Ingenia 3.0-T MRI scanner (Royal Philips, Amsterdam, Netherlands), according to the methodology described in our previous study (17). Images of 3 coronal sections of the kidneys were acquired at each examination, then the right kidney was evaluated, unless large cysts or other anatomical abnormalities necessitated assessment of the left kidney to avoid artifacts. Imaging parameters were the same as those noted in our previous study (flip angle 50°, echo time 12 acquisitions [4.92–31.98 ms], repetition time 172 ms, slice thickness 5 mm). T_2_* values were analyzed using two methods, as shown following.

#### 2.5.1 Twelve-layer concentric object method

The renal parenchyma was semi-automatically divided into 12 layers of equal thickness using MATLAB R2020b (The MathWorks, Natick, MA, USA), with the mean value from the first to third layers utilized as an indicator of cortical oxygenation. TLCO image analysis was conducted using a standardized pipeline implemented in MATLAB, based on previously reported methods (11, 19).

#### 2.5.2 Region of interest method

ROI was manually determined within the renal cortex using coronal T_2_-weighted images, encompassing the entire anatomically identifiable cortical region, with regions containing cysts, mass lesions, or stones avoided. In advanced CKD cases, the cortico-medullary boundary was difficult to delineate and the ROI was placed in the subcapsular parenchyma (12). ROI placement followed predefined criteria and was performed in a consistent manner across all subjects, avoiding large vessels and imaging artifacts. In the *post-hoc* analysis, medullary oxygenation was not evaluated and apparent diffusion coefficient (ADC) data from diffusion-weighted imaging (DWI) were not analyzed.

### 2.6 Statistical analysis

Changes in RI following Cana administration were evaluated, while the association with kidney tissue oxygenation was assessed using T_2_* mapping. Changes in RI are expressed as ΔRI, while changes in T_2_* from Day 0 to Day 1 and from Day 0 to Day 5 are expressed as ΔT_2_*(D1–D0) and ΔT_2_*(D5–D0), respectively. Analyses were conducted using data from our previous study regarding the effects of Cana on kidney oxygenation and metabolism (17). Baseline characteristics of the 13 subjects were summarized using median values and interquartile range (IQR) for continuous variables, and as percentage for categorical variables. In our prior study, T_2_* values were analyzed as geometric means, estimated using generalized least squares, while RI and T_2_* values in the present *post-hoc* analysis were obtained using TLCO and cortical ROI methods on Day 0, Day 1, and Day 5, and are presented as median values (IQR). A Wilcoxon signed-rank test was used to compare RI between Day 0 and Day 5, and T_2_* values between Day 0 and Day 1, and Day 0 and Day 5. Effect sizes (r) for the Wilcoxon signed-rank test were calculated as Z/√n. To account for multiple comparisons, Bonferroni correction was applied to comparisons of T_2_* values, and the level of significance for these paired comparisons was set at *P*< 0.025. Associations of ΔRI with ΔT_2_* (D1–D0) and ΔT_2_* (D5–D0), as well as changes in other renal or metabolic parameters, including estimated glomerular filtration rate (ΔeGFR), fasting plasma glucose (ΔFPG), urinary albumin excretion (ΔUAE), and body weight (ΔBW), were assessed with Spearman’s rank correlation coefficient. Correlation analyses were considered exploratory and were not adjusted for multiple comparisons. All statistical analyses were performed using EZR (v. 1.54) and R (R Foundation for Statistical Computing, Vienna, Austria). EZR is a graphical user interface for R developed by Dr. Yoshinobu Kanda, of Saitama Medical Center, Jichi Medical University, Saitama, Japan (20). The level of significance was set at *P*< 0.05, except for paired comparisons where Bonferroni correction was applied (*P*< 0.025).

## 3 Results

Of 14 enrolled patients, one subject was excluded from the present *post-hoc* analysis because of severe kidney atrophy, which prevented determination of T_2_* values using the TLCO method, thus analyses of 13 patients were performed (**Supplementary Figure 2**). Their clinical characteristics are summarized in **Table 1**. Median age was 63.0 years (interquartile range: 59.0-72.0), body mass index (BMI) was 24.5 (22.5–27.2) kg/m², HbA1c was 7.1 (6.9–7.8) %, eGFR was 66.2 (47.4–77.7) mL/min/1.73 m², and UAE was 15.1 (5.1–492.8) mg/day. Representative T2-weight, T_2_* map, and quantitative evaluation images are presented in **Supplementary Figure S3**.

**Table 1:** Clinical characteristics of participants.

Variables	Overall (n=13)
Age, years (range)	63.0 (59.0-72.0)
Male/female	10/3 (77/23)
Body mass index, kg/m^2^	24.5 (22.5-27.2)
Systolic blood pressure, mmHg	126.0 (111-132)
Hemoglobin, g/dL	14.3 (13.2-15.4)
Albumin, g/dL	3.9 (3.9-4.1)
Blood urea nitrogen, mg/dL	14.0 (13.0-17.0)
Creatinine, mg/dL	0.89 (0.73-1.28)
Estimated GFR, mL/min/1.73 m^2^	66.2 (47.4-77.7)
Fasting plasma glucose, mg/dL	120 (106-161)
HbA1c, %	7.1 (6.9-7.8)
Glycated albumin, %	18.6 (16.3-19.6)
Urinary albumin, mg/day	15.1 (5.1-492.8)
Medications	
DPP-4 inhibitors (%)	8 (62)
Metformin (%)	6 (46)
GLP-1 receptor agonists (%)	1 (8)
Glinide (%)	1 (8)
Insulin (%)	1 (8)
ACE inhibitor or ARB (%)	6 (46)
Statin (%)	10 (77)

Data are presented as median (interquartile range) or number (%), unless otherwise noted.

**Figure 1:**
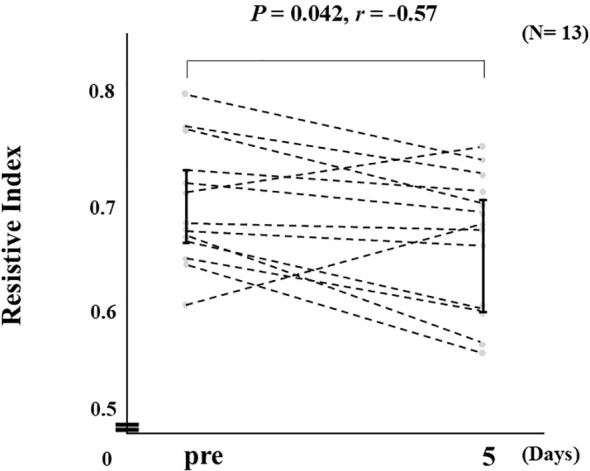
Changes in RI from Pre (screening) to Day 5 following canagliflozin (Cana) administration starting on Day 1 (n=13). Data are presented as individual values, and error bars represent the interquartile range (IQR).

**Figure 2:**
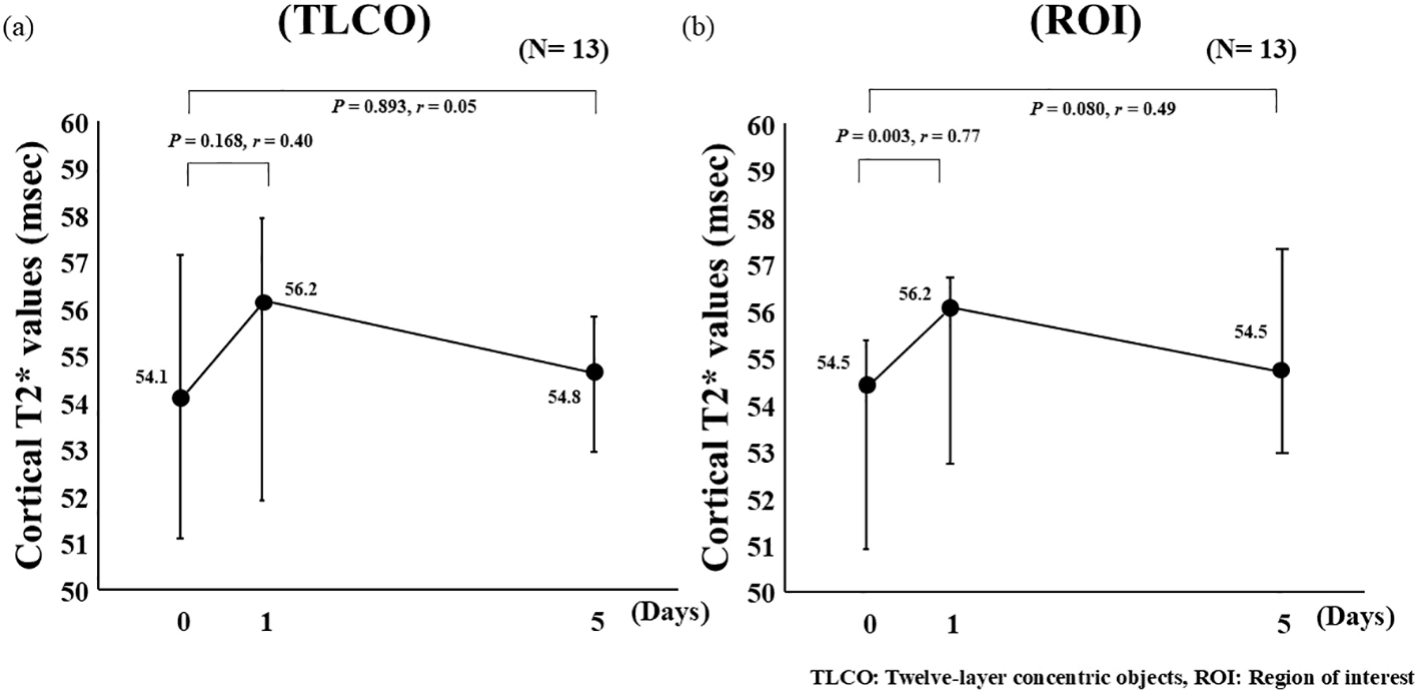
Changes in cortical T_2_* values from day 0 (baseline) to day 1 (time of initial single-dose cana administration) and day 5 (after 5 consecutive days of treatment), assessed using **(a)** TLCO and **(b)** ROI. TLCO, twelve-layer concentric object; ROI, region of interest. Data are presented as median [interquartile range (IQR)], and error bars represent the IQR.

**Figure 3:**
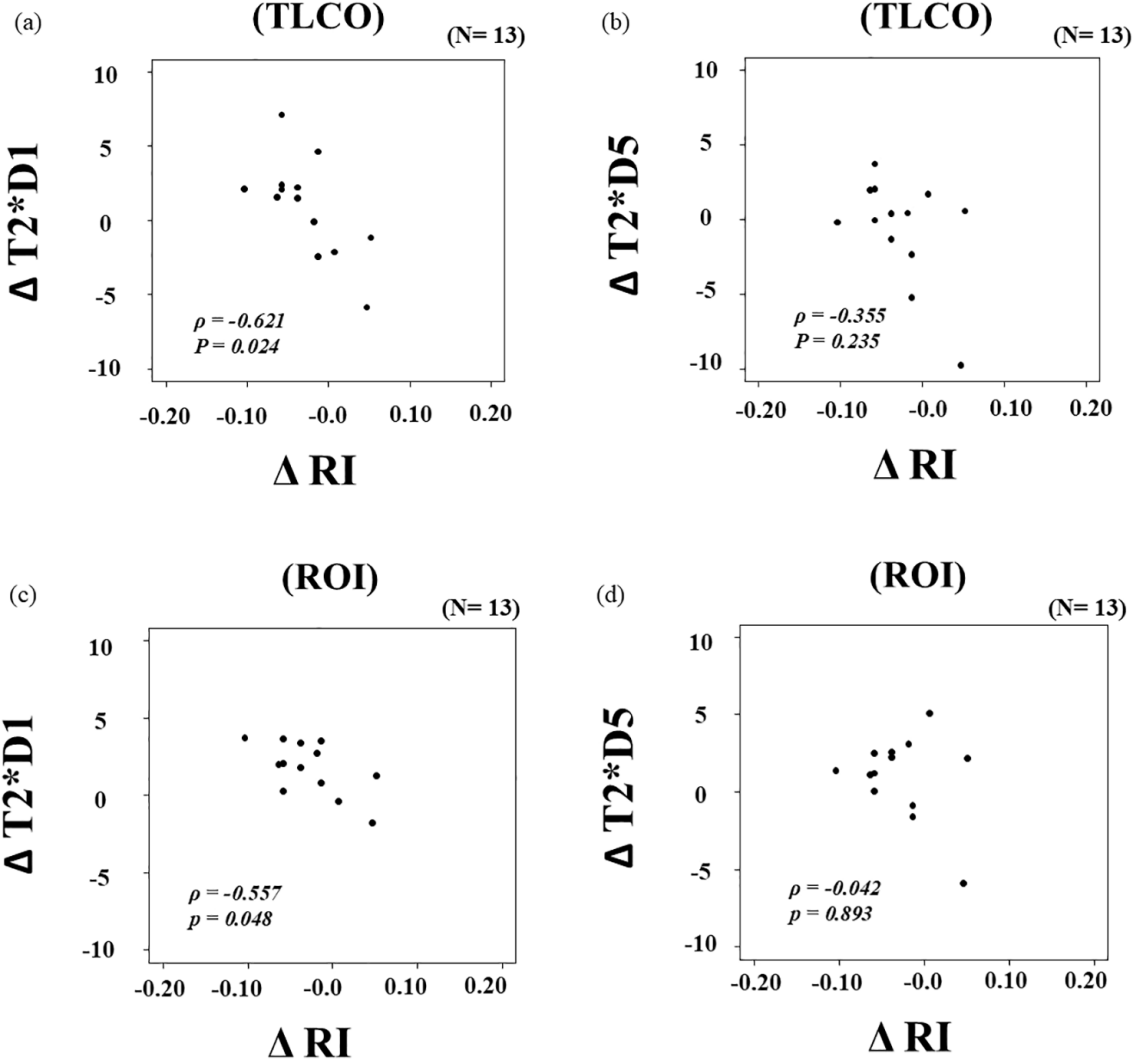
Associations of changes in resistive index (ΔRI) and T_2_* values (ΔT_2_*) using TLCO **(a, b)** and ROI **(c, d)**. TLCO, twelve-layer concentric object; ROI, region of interest.Changes in RI from D0 to D1 or D5 are expressed as ΔRI, while changes in T_2_* from D0 to D1 are expressed as ΔT_2_* (D1–D0) and from D0 to D5 as ΔT_2_* (D5–D0).

Administration of Cana resulted in significantly decreased RI on Day 5 as compared to the screening measurement (median values; *p* = 0.042, *r* = -0.57) (**Figure 1**). Analysis of BOLD MRI findings using ROI showed that Cana administration was associated with a significant increase in T_2_* values on Day 1 (D0: 54.5 [50.8–55.5], D1: 56.2 [52.7–56.4], *p* = 0.003, *r* = 0.77), whereas no significant change was observed at Day 5 (D5: 54.5 [53.0–57.2], *p* = 0.080, *r* = 0.49) (**Figure 2**). A significant negative correlation was observed between ΔRI and ΔT_2_* (D1–D0) in both TLCO (*ρ* = -0.621, *p* = 0.024) and ROI (*ρ* = -0.557, *p* = 0.048) results (**Figures 3A, C**), whereas no significant correlation between ΔRI and ΔT_2_* (D5–D0) was indicated (**Figures 3B, D**). Changes in clinical parameters before and following Cana administration, as well as correlations between ΔRI and changes in various clinical parameters are summarized in **Tables 2** and **3**.

**Table 2:** Changes in various clinical parameters before and after canagliflozin administration.

Parameter	Pre	Day 0	Day 1	Day 2	Day 5
eGFR (mL/min/1.73 m^2^)		66.2 (47.4-66.2)		57.7 (41.7-66.8) *p* = 0.002 vs Day 0	56.4 (42.3-67.6) *p* = 0.001 vs Day 0
Erythropoietin (IU/L)		8.30 (6.10-10.20)			8.1 (6.7-10.8) *p* = 0.845 vs Day 0
Fasting plasma glucose (FPG) (mg/dL)		120 (106-161)		108 (103-132) *p* = 0.005 vs Day 0	110 (96-127) *p* = 0.004 vs Day 0
HbA1c (%)		7.10 (6.90-7.80)			7.00 (6.80-7.90) *p* = 0.125 vs Day 0
Glycated albumin (GA) (%)		18.6 (16.3-19.6)			18.0 (15.8-19.2) *p* = 0.002 vs Day 0
Urinary albumin (mg/day)		15.1 (5.1-492.8)		8.0 (6.7-365.7) *p* = 0.052 vs Day 0	14.2 (6.5-356.6) *p* = 0.011 vs Day 0
Body weight (kg)		64.7 (59.2, 77.8)		64.4 (58.6, 76.6) *p* = 0.004 vs Day0	63.9 (57.9, 76.5) *p* = 0.002 vs Day 0
T2* (TLCO)		54.1 (51.2, 57.2)	56.2 (51.6, 57.8) *p* = 0.168 vs Day 0		54.8 (52.8, 55.8) *p* = 0.893 vs Day 0
T2* (ROI)		54.5 (50.8, 55.5)	56.2 (52.7, 56.4) *p* = 0.003 vs Day 0		54.5 (53.0, 57.2) *p* = 0.080 vs Day 0
Resistive index	0.675 (0.665, 0.720)				0.680 (0.610, 0.705) *p* = 0.042 vs Day 0

Data are presented as median (interquartile). *p* values were obtained using a Wilcoxon signed-rank test. TLCO, twelve-layer concentric object; ROI, region of interest.

**Table 3:** Association between changes in resistive index (ΔRI) and various clinical parameters before and after canagliflozin administration.

Parameter	ρ	*p*
ΔeGFR *D2	0.352	0.238
ΔeGFR *D5	0.380	0.201
ΔFPG *D2	0.477	0.099
ΔFPG *D5	0.388	0.190
ΔUrinary albumin *D2	-0.047	0.879
ΔUrinary albumin *D5	0.307	0.307
ΔBody weight *D2	0.518	0.070
ΔBody weight *D5	0.144	0.640
ΔT_2_* D1 (TLCO)	-0.621	0.024
ΔT_2_* D5 (TLCO)	-0.355	0.235
ΔT_2_* D1 (ROI)	-0.557	0.048
ΔT_2_* D5 (ROI)	-0.042	0.893

Data are presented as median (interquartile). *p* values were obtained using Spearman’s correlation coefficient.

*D1, Day 1-Day 0; *D2, Day 2-Day 0; *D5, Day 5-Day 0.

TLCO, twelve-layer concentric object; ROI, region of interest.

## 4 Discussion

The results of 13 patients with T2D were analyzed in this study. Cana significantly decreased RI. Using the ROI method, T_2_* values were significantly increased on Day 1 compared with Day 0 (*p* = 0.003, *r* = 0.77). In contrast, the increase on Day 5 did not reach statistical significance after Bonferroni correction, although the effect size remained moderate (*p* = 0.080, *r* = 0.49). Furthermore, there was a significant negative correlation between ΔRI and ΔT_2_* (D1–D0) noted in both TLCO (*p* = 0.024) and ROI (*p* = 0.048) findings, whereas no significant correlation was found between ΔRI and ΔT_2_* (D5–D0).

RI is generally regarded as an indicator of intrarenal peripheral vascular resistance. Indeed, in db/db mice, RI has been shown to be significantly higher than in wild-type controls (21), while increases in RI have been observed in patients with T2D in association with progression of diabetic nephropathy and inversely correlated with creatinine clearance (10). More recent studies have shown that RI is influenced by systemic vascular compliance and cardiac output (22, 23), as well as overall systemic hemodynamic conditions (6, 24, 25). In addition, RI serves as a prognostic marker. In patients with heart failure, elevated RI has been reported to be correlated with disease severity and adverse outcomes (9), and in DKD patients with normoalbuminuria it is used an indicator of future progression of nephropathy (26). RI may also reflect reversible renal hemodynamic changes, as suggested by results showing reduction in RI following bariatric surgery in patients with severe obesity, who are generally characterized by glomerular hyperfiltration and increased renal plasma flow (27, 28).

RI improvement observed in the present patients following administration of Cana is likely attributable not only to correction of glomerular hyperfiltration, as the findings also indicate that it enhanced systemic vascular compliance, improved glycemic control, and attenuated oxidative stress. Notably, a previous study reported that short-term administration (2 days) of dapagliflozin in patients with T2D and preserved kidney function significantly improved RI, as well as endothelial function and aortic pulse wave velocity (13). Importantly, the present results indicate that short-term Cana administration significantly improves RI even in patients with T2D, including those with impaired kidney function.

Our previous study found that Cana improves kidney cortical oxygenation (17). This effect is related to glucose handling in the proximal tubules, where its reabsorption is an energy- and oxygen-consuming process (29). In this pathway, intracellular glucose is transported into the bloodstream via basolateral glucose transporter type 2, a transmembrane carrier protein, in a concentration-dependent manner, a process that requires ATP synthesis and thereby consumes oxygen (30). By inhibiting glucose reabsorption, Cana is thought to reduce kidney oxygen demand.

The present findings demonstrated a significant negative correlation between reduction in RI and improvement in kidney cortical oxygenation (D1–D0). It is considered that improved cortical oxygenation may contribute to reduction in RI by attenuating oxidative stress, restoring endothelial nitric oxide (NO) bioavailability, promoting arteriolar dilation, and normalizing renal plasma flow (13, 31). Moreover, Cana might further enhance RI and cortical oxygenation by improving systemic hemodynamics and modulation of cardio-renal interactions via afferent pathways, including the sympathetic nervous system (13, 25). However, no significant correlation was observed between ΔRI and ΔT_2_* (D5–D0).

The attenuation of the increase in T_2_* values observed on Day 1 by Day 5 may be explained by the balance between oxygen consumption and oxygen supply. SGLT2 inhibition reduces tubular oxygen consumption in the early phase by inhibiting sodium reabsorption in the proximal tubule. In contrast, particularly in type 1 diabetes, tubuloglomerular feedback induces afferent arteriolar vasoconstriction. In addition, in patients with type 2 diabetes, SGLT2 inhibitors have been suggested to induce efferent arteriolar dilation (32). These hemodynamic changes may contribute to a reduction in renal blood flow and, consequently, decreased oxygen supply.

The present study included relatively older patients with type 2 diabetes compared with previous studies (14, 15), suggesting that structural vascular changes, such as afferent arteriolar hyalinosis, may have already been present. Under such conditions, the combined effects of afferent arteriolar constriction and efferent arteriolar dilation may have led to a more pronounced reduction in renal blood flow following canagliflozin administration. Consistent with this interpretation, a relatively large initial dip was observed in our study. As a result, the early improvement in cortical oxygenation observed at Day 1 may have been offset by a subsequent decrease in oxygen supply, thereby limiting further increases in T_2_* at Day 5. These findings, consistent with previous reports (14), suggest a temporal dissociation between hemodynamic changes in GFR and tissue oxygenation tissue oxygenation responses following SGLT2 inhibition, with early metabolic effects and later hemodynamic influences contributing to the observed pattern. Further studies are needed to clarify the long-term effects of SGLT2 inhibitors on kidney oxygenation in similar patient populations.

This study has some limitations. First, the number of patients examined was relatively small and there was no control group included. To confirm the correlation between improvements in kidney oxygenation and RI following Cana administration observed in the present results, additional studies with a larger sample size and appropriate control group are needed. Second, kidney oxygenation was evaluated in the subjects under inpatient conditions, while RI was assessed during screening in an outpatient setting, which may have introduced variability due to differences in the environments. Finally, direct measurements were not performed to determine oxidative stress, glomerular filtration rate, or renal perfusion. In addition, given the exploratory *post hoc* nature of the analysis and the small sample size, statistical power was limited, and more advanced modeling approaches for repeated measurements were not used. Therefore, the findings should be interpreted with caution.

It is suggested that the inverse correlation between changes in RI and T_2_* values indicates a mechanistic link between hemodynamic changes and improved oxygenation induced by Cana. The effects of SGLT2 inhibitors may contribute to improved long-term kidney outcomes in affected individuals.

## Data Availability

The original contributions presented in the study are included in the article/**Supplementary Material**. Further inquiries can be directed to the corresponding author.

## References

[1] Perkovic V Jardine MJ Neal B Bompoint S Heerspink HJL Charytan DM . Canagliflozin and renal outcomes in type 2 diabetes and nephropathy. N Engl J Med. (2019) 380:2295–306. doi: 10.1056/nejmoa1811744. PMID: 30990260

[2] Neal B Perkovic V Mahaffey KW de Zeeuw D Fulcher G Erondu N . Canagliflozin and cardiovascular and renal events in type 2 diabetes. N Engl J Med. (2017) 377:644–57. doi: 10.1056/nejmoa1611925. PMID: 28605608

[3] Wiviott SD Raz I Bonaca MP Mosenzon O Kato ET Cahn A . Dapagliflozin and cardiovascular outcomes in type 2 diabetes. N Engl J Med. (2019) 380:347–57. doi: 10.1056/nejmoa1812389. PMID: 30415602

[4] Wanner C Inzucchi SE Lachin JM Fitchett D von Eynatten M Mattheus M . Empagliflozin and progression of kidney disease in type 2 diabetes. N Engl J Med. (2016) 375:323–34. doi: 10.1056/nejmoa1515920. PMID: 27299675

[5] Tomita I Kume S Sugahara S Osawa N Yamahara K Yasuda-Yamahara M . SGLT2 inhibition mediates protection from diabetic kidney disease by promoting ketone body-induced mTORC1 inhibition. Cell Metab. (2020) 32:404–19.e6. doi: 10.1016/j.cmet.2020.06.020. PMID: 32726607

[6] Di Nicolò P Granata A . Renal intraparenchymal resistive index: the ultrasonographic answer to many clinical questions. J Nephrol. (2019) 32:527–38. doi: 10.1007/s40620-018-00567-x. PMID: 30539416

[7] Bruno RM Daghini E Landini L Versari D Salvati A Santini E . Dynamic evaluation of renal resistive index in normoalbuminuric patients with newly diagnosed hypertension or type 2 diabetes. Diabetologia. (2011) 54:2430–9. doi: 10.1007/s00125-011-2148-y. PMID: 21499674

[8] Veglio F Frascisco M Melchio R Provera E Rabbia F Oliva S . Assessment of renal resistance index after captopril test by Doppler in essential and renovascular hypertension. Kidney Int. (1995) 48:1611–6. doi: 10.1038/ki.1995.455. PMID: 8544422

[9] Ciccone MM Iacoviello M Gesualdo L Puzzovivo A Antoncecchi V Doronzo A . The renal arterial resistance index: a marker of renal function with an independent and incremental role in predicting heart failure progression. Eur J Heart Fail. (2014) 16:210–6. doi: 10.1002/ejhf.34. PMID: 24464953

[10] Ishimura E Nishizawa Y Kawagishi T Okuno Y Kogawa K Fukumoto S . Intrarenal hemodynamic abnormalities in diabetic nephropathy measured by duplex Doppler sonography. Kidney Int. (1997) 51:1920–7. doi: 10.1038/ki.1997.261. PMID: 9186883

[11] Pruijm M Milani B Pivin E Podhajska A Vogt B Stuber M . Reduced cortical oxygenation predicts a progressive decline of renal function in patients with chronic kidney disease. Kidney Int. (2018) 93:932–40. doi: 10.1016/j.kint.2017.10.020. PMID: 29325997

[12] Sugiyama K Inoue T Kozawa E Ishikawa M Shimada A Kobayashi N . Reduced oxygenation but not fibrosis defined by functional magnetic resonance imaging predicts the long-term progression of chronic kidney disease. Nephrol Dial Transplant. (2020) 35:964–70. doi: 10.1093/ndt/gfy324. PMID: 30418615

[13] Solini A Giannini L Seghieri M Vitolo E Taddei S Ghiadoni L . Dapagliflozin acutely improves endothelial dysfunction, reduces aortic stiffness and renal resistive index in type 2 diabetic patients: a pilot study. Cardiovasc Diabetol. (2017) 16:138. doi: 10.1186/s12933-017-0621-8. PMID: 29061124 PMC5654086

[14] Laursen JC Søndergaard-Heinrich N de Melo JML Haddock B Rasmussen IKB Safavimanesh F . Acute effects of dapagliflozin on renal oxygenation and perfusion in type 1 diabetes with albuminuria: a randomized, double-blind, placebo-controlled crossover trial. EClinicalMedicine. (2021) 37:100895. doi: 10.1016/j.eclinm.2021.100895. PMID: 34386735 PMC8343250

[15] Zhou S Zhang Y Wang T Huang S Gong S Wang J . Canagliflozin could improve the levels of renal oxygenation in newly diagnosed type 2 diabetes patients with normal renal function. Diabetes Metab. (2021) 47:101274. doi: 10.1016/j.diabet.2021.101274. PMID: 34481963

[16] Gullaksen S Vernstrøm L Sørensen SS Ringgaard S Laustsen C Funck KL . Separate and combined effects of semaglutide and empagliflozin on kidney oxygenation and perfusion in people with type 2 diabetes: a randomized trial. Diabetologia. (2023) 66:813–25. doi: 10.1007/s00125-023-05876-w. PMID: 36746803

[17] Mori K Inoue T Machiba Y Uedono H Nakatani S Ishikawa M . Effects of canagliflozin on kidney oxygenation evaluated using blood oxygenation level-dependent MRI in patients with type 2 diabetes. Front Endocrinol (Lausanne). (2024) 15:1451671. doi: 10.3389/fendo.2024.1451671. PMID: 39280006 PMC11393780

[18] Iijima H Kifuji T Maruyama N Inagaki N . Pharmacokinetics, pharmacodynamics, and safety of canagliflozin in Japanese patients with type 2 diabetes mellitus. Adv Ther. (2015) 32:768–82. doi: 10.1007/s12325-015-0234-0. PMID: 26280756 PMC4569680

[19] Inoue T Kozawa E Ishikawa M Fukaya D Amano H Watanabe Y . Comparison of multiparametric magnetic resonance imaging sequences with laboratory parameters for prognosticating renal function in chronic kidney disease. Sci Rep. (2021) 11:22129. doi: 10.1038/s41598-021-01147-z. PMID: 34764322 PMC8586015

[20] Kanda Y . Investigation of the freely available easy-to-use software 'EZR' for medical statistics. Bone Marrow Transplant. (2013) 48:452–8. doi: 10.1038/bmt.2012.244. PMID: 23208313 PMC3590441

[21] Faita F Di Lascio N Rossi C Kusmic C Solini A . Ultrasonographic characterization of the db/db mouse: an animal model of metabolic abnormalities. J Diabetes Res. (2018) 2018:4561309. doi: 10.1016/j.artres.2017.10.095. PMID: 29707583 PMC5863337

[22] Di Nicolò P Granata A . Renal resistive index: not only kidney. Clin Exp Nephrol. (2017) 21:359–66. doi: 10.1007/s10157-016-1323-3. PMID: 27530995

[23] Krumme B . Renal Doppler sonography--update in clinical nephrology. Nephron Clin Pract. (2006) 103:c24–8. doi: 10.1159/000090605. PMID: 16543752

[24] Heine GH Reichart B Ulrich C Köhler H Girndt M . Do ultrasound renal resistance indices reflect systemic rather than renal vascular damage in chronic kidney disease? Nephrol Dial Transplant. (2007) 22:163–70. doi: 10.1093/ndt/gfl484. PMID: 16936334

[25] O'Neill WC . Renal resistive index: a case of mistaken identity. Hypertension. (2014) 64:915–7. doi: 10.1161/HYPERTENSIONAHA.114.04183, PMID: 25156171

[26] Masulli M Mancini M Liuzzi R Daniele S Mainenti PP Vergara E . Measurement of the intrarenal arterial resistance index for the identification and prediction of diabetic nephropathy. Nutr Metab Cardiovasc Dis. (2009) 19:358–64. doi: 10.1016/j.numecd.2008.07.003. PMID: 18805683

[27] Vitolo E Santini E Salvati A Volterrani D Duce V Bruno RM . Metabolic and hormonal determinants of glomerular filtration rate and renal hemodynamics in severely obese individuals. Obes Facts. (2016) 9:310–20. doi: 10.1159/000446965. PMID: 27701167 PMC5644791

[28] Solini A Seghieri M Santini E Giannini L Biancalana E Taddei S . Renal resistive index predicts post-bariatric surgery renal outcome in nondiabetic individuals with severe obesity. Obes (Silver Spring). (2019) 27:68–74. doi: 10.1002/oby.22355. PMID: 30516353

[29] Palmer BF Clegg DJ . Kidney-protective effects of SGLT2 inhibitors. Clin J Am Soc Nephrol. (2023) 18:279–89. doi: 10.2215/cjn.09380822. PMID: 36220189 PMC10103214

[30] O'Neill J Fasching A Pihl L Patinha D Franzén S Palm F . Acute SGLT inhibition normalizes O2 tension in the renal cortex but causes hypoxia in the renal medulla in anaesthetized control and diabetic rats. Am J Physiol Renal Physiol. (2015) 309:F227–34. doi: 10.1152/ajprenal.00689.2014, PMID: 26041448

[31] Lin B Koibuchi N Hasegawa Y Sueta D Toyama K Uekawa K . Glycemic control with empagliflozin, a novel selective SGLT2 inhibitor, ameliorates cardiovascular injury and cognitive dysfunction in obese and type 2 diabetic mice. Cardiovasc Diabetol. (2014) 13:148. doi: 10.1186/s12933-014-0148-1. PMID: 25344694 PMC4219031

[32] León-Jiménez D Sridhar VS López-Mendoza M Scholtes RA Schmieder RE Cherney DZI . Kidney hemodynamic effects of sodium-glucose cotransporter 2 inhibitors in diabetes: physiology and clinical implications. Clin Kidney J. (2025) 18:sfae370. doi: 10.1093/ckj/sfae370, PMID: 40008354 PMC11852268

