# Supplementary figures and images for: Effects of canagliflozin on kidney resistive index and oxygenation in patients with type 2 diabetes: findings obtained with ultrasonography and blood oxygenation level-dependent MRI
Source: Front Clin Diabetes Healthc. 2026 Apr 29;7:1733806. doi: 10.3389/fcdhc.2026.1733806 (PMC13167403; doi:10.3389/fcdhc.2026.1733806)

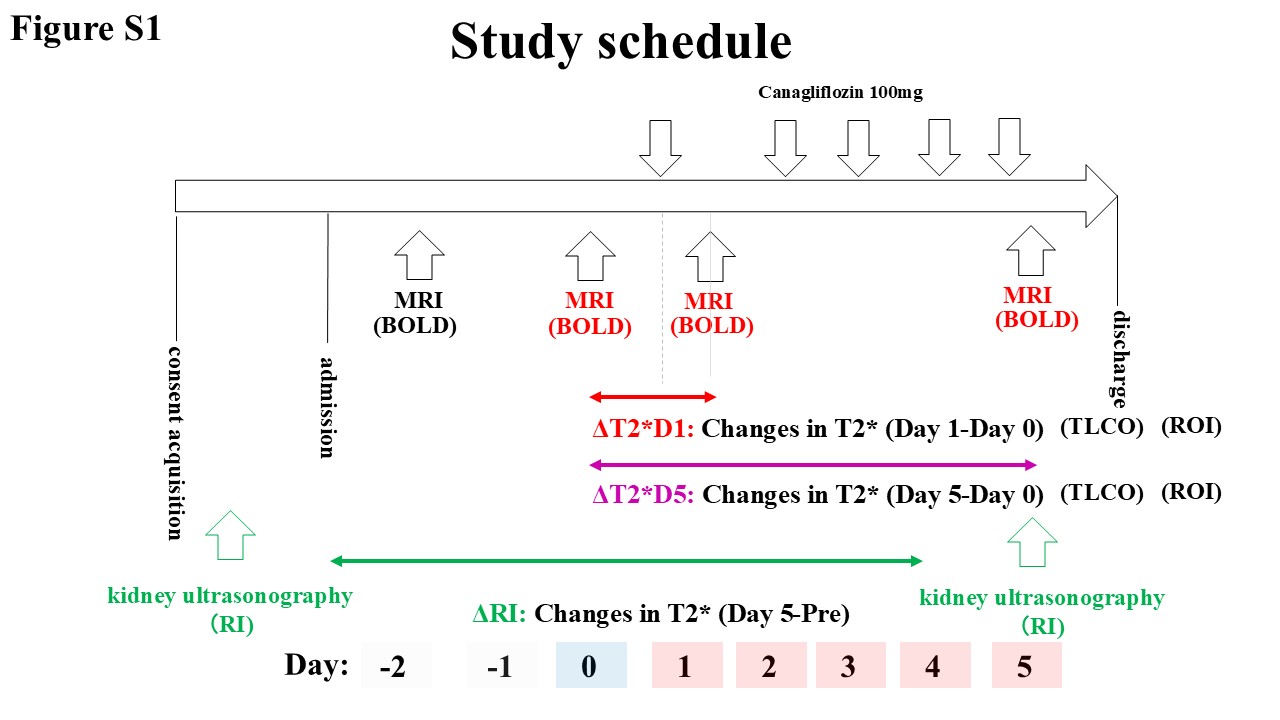

Supplement: Supplementary file 1 [file Image1.jpeg]

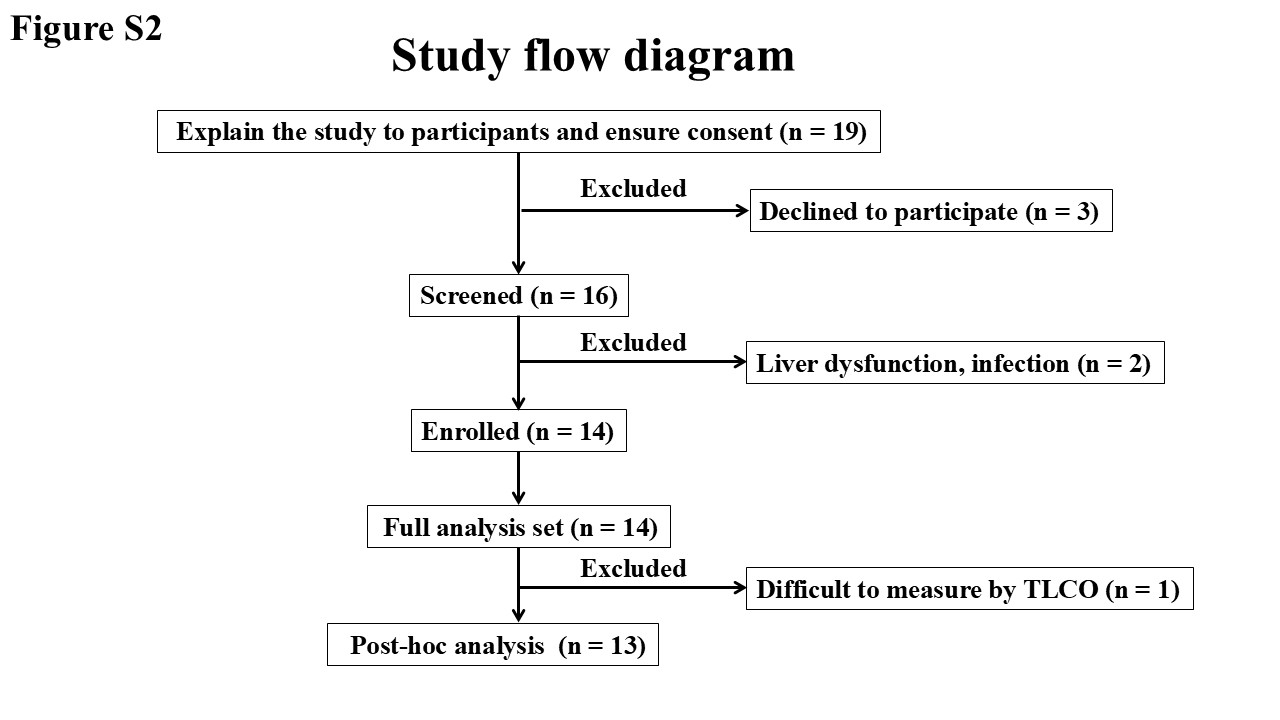

Supplement: Supplementary file 2 [file Image2.jpeg]

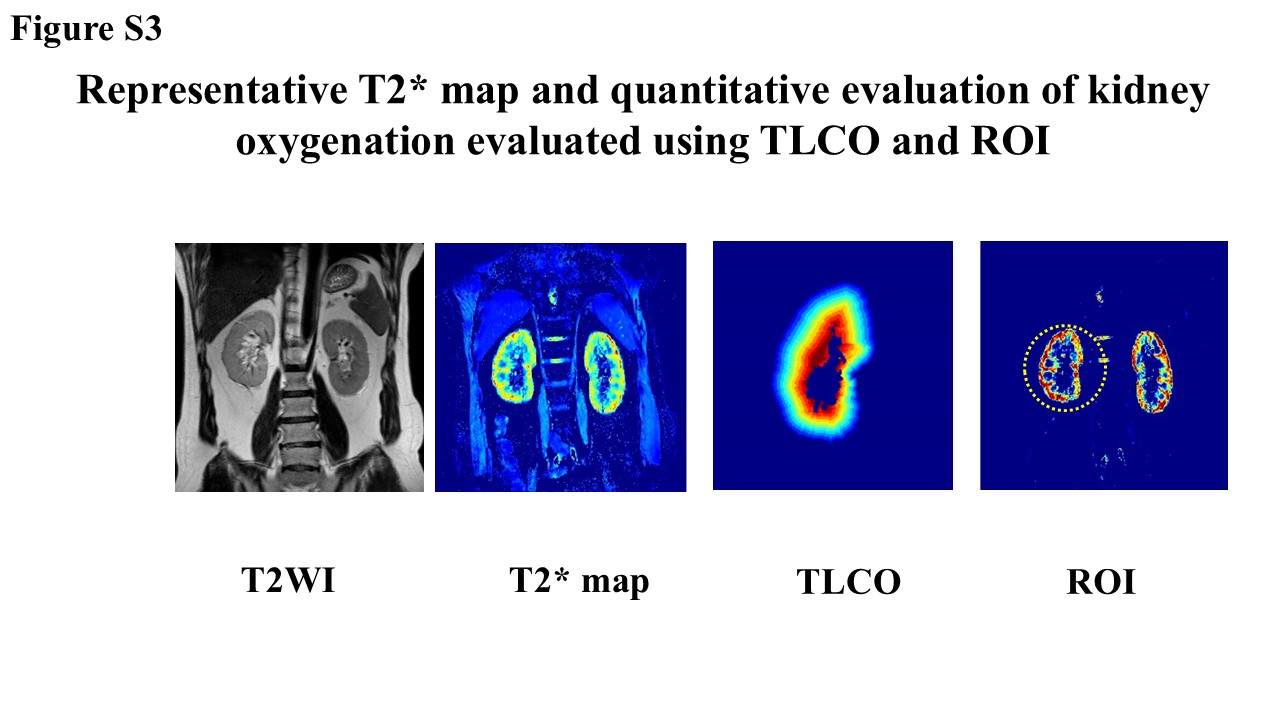

Supplement: Supplementary file 3 [file Image3.jpeg]
